# Mesenchymal Stem Cells: A Therapeutic Approach in Fertility Restoration in Premature Ovarian Insufficiency

**DOI:** 10.1007/s12015-025-10944-2

**Published:** 2025-08-01

**Authors:** Nourhan Hassan, Donia Mohamed Hussein, Fady Ashraf Malak, Mazen Ashraf Abdelaziz, Michael Ibrahim Boushra, Walid Shaalan, Emad M. Elzayat

**Affiliations:** 1 Biotechnology Department, Faculty of Science, Cairo University, Giza, 12613 Egypt; 2 Biotechnology/Biomolecular Chemistry Program, Faculty of Science, Cairo University, Giza, 12613 Egypt; 3 Department of Gynecology and Obstetrics, Heidelberg University Hospital, Im Neuenheimer Feld 440, 69120 Heidelberg, Germany

**Keywords:** Premature ovarian dysfunction, Mesenchymal stem cells, Fertility restoration, Exosomes, Extracellular vesicles, Ovarian rejuvenation, Regenerative medicine

## Abstract

Primary ovarian insufficiency (POI) represents the cessation of ovarian function before age 40 due to follicular depletion or dysfunction. Affecting 1% of women, POI causes infertility and systemic health complications. Current treatments focus on symptom management rather than restoring ovarian function. Mesenchymal stem cells (MSCs) offer promising regenerative potential through paracrine activity, immunomodulation, and tissue repair mechanisms. MSCs from various sources have demonstrated the ability to improve ovarian function, increase follicular survival, and restore hormone production in preclinical models. MSC-derived extracellular vesicles are emerging as cell-free alternatives with similar therapeutic effects. This review examines MSC mechanisms in ovarian restoration, evaluates current evidence, and discusses challenges in clinical translation for POI treatment.

**Graphical Abstract:**

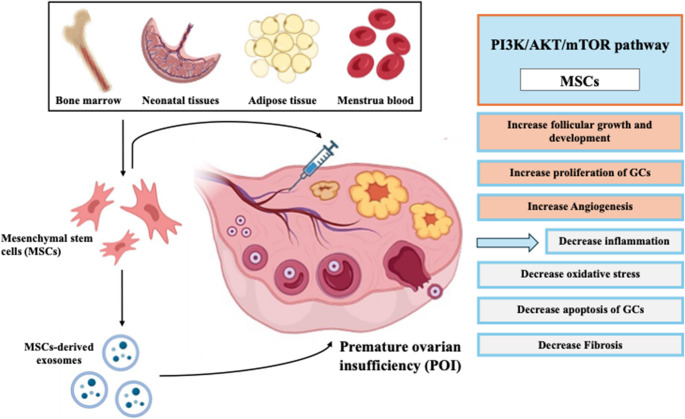

## Introduction

Globally, reproductive aging is increasing and is closely linked to aging in general [1]. Premature ovarian insufficiency (POI) is a severe form of reproductive aging that has primarily had an unexplained origin up to this point, which has limited its therapeutic applicability and resulted in significant personal and financial expenses [2, 3]. Before the age of 40, POI is a clinical illness marked by biochemical proof of ovarian insufficiency and loss of ovarian function, which is indicated by irregular menstruation periods. One to three% of women in the general population suffer from POI [3]. The incidence of POI is age-specific; it affects 1 in 250 women by the age of 35 and 1 in 100 by the age of 40 [4]. The clinical sequelae of POI extend far beyond infertility, impacting neurological, psychological (including increased risk of depression and anxiety), cardiovascular (due to estrogen deficiency), sexual, and bone health (leading to osteoporosis), thereby posing multifaceted challenges for both patients and healthcare providers (HCPs) [5]. These multifaceted complications pose significant challenges for both patients and healthcare providers [6].

Regenerative medicine shows promise for treating pathological illnesses that currently lack effective cures [7]. Stem cells form the foundation of regenerative medicine approaches. These cells are derived from multicellular organisms and possess the ability to differentiate into several cell types (potency) while also producing more of their own kind (self-renewal) [8, 9]. They represent groups of unspecialized cells with the capacity to develop into distinct cellular subtypes.

Mesenchymal stem cells (MSCs) have garnered significant interest in cell therapy and regenerative medicine due to their self-renewal capacity, differentiation potential, and immunomodulatory properties [10]. MSCs primarily exert their therapeutic effects through paracrine mechanisms. They secrete bioactive molecules including growth factors, cytokines, chemokines, and extracellular vesicles that collectively modulate the tissue microenvironment, reduce inflammation, inhibit apoptosis, promote angiogenesis, and stimulate endogenous repair processes [11].

The application of MSCs in POI treatment has shown considerable promise in preclinical studies using various animal models. MSC transplantation has been demonstrated to improve ovarian reserve, enhance follicular development, restore hormone production, and even lead to successful pregnancies in these models [12, 13]. These beneficial effects are attributed to MSCs’ ability to home to damaged ovarian tissue, reduce local inflammation, promote survival of existing follicles, stimulate angiogenesis, and potentially activate dormant primordial follicles [10, 14–17].

POI can result from various causes including genetic factors (e.g., Turner syndrome, fragile X premutation, mutations in genes involved in follicular development), autoimmune disorders, iatrogenic causes (chemotherapy, radiotherapy, ovarian surgery), environmental factors, and idiopathic causes which still account for the majority of cases [3, 18]. This review provides a comprehensive overview of the current understanding and therapeutic potential of MSCs in restoring fertility and ovarian function in women with POI. We explore the pathophysiology of POI, the diverse regenerative mechanisms of MSCs, and their potential to rejuvenate ovarian function. Additionally, we examine the existing preclinical and emerging clinical evidence supporting MSC-based therapies, discuss current challenges in their application, and highlight future directions for research.

### Pathophysiology and Etiology of Premature Ovarian Insufficiency

**Fig. 1:**
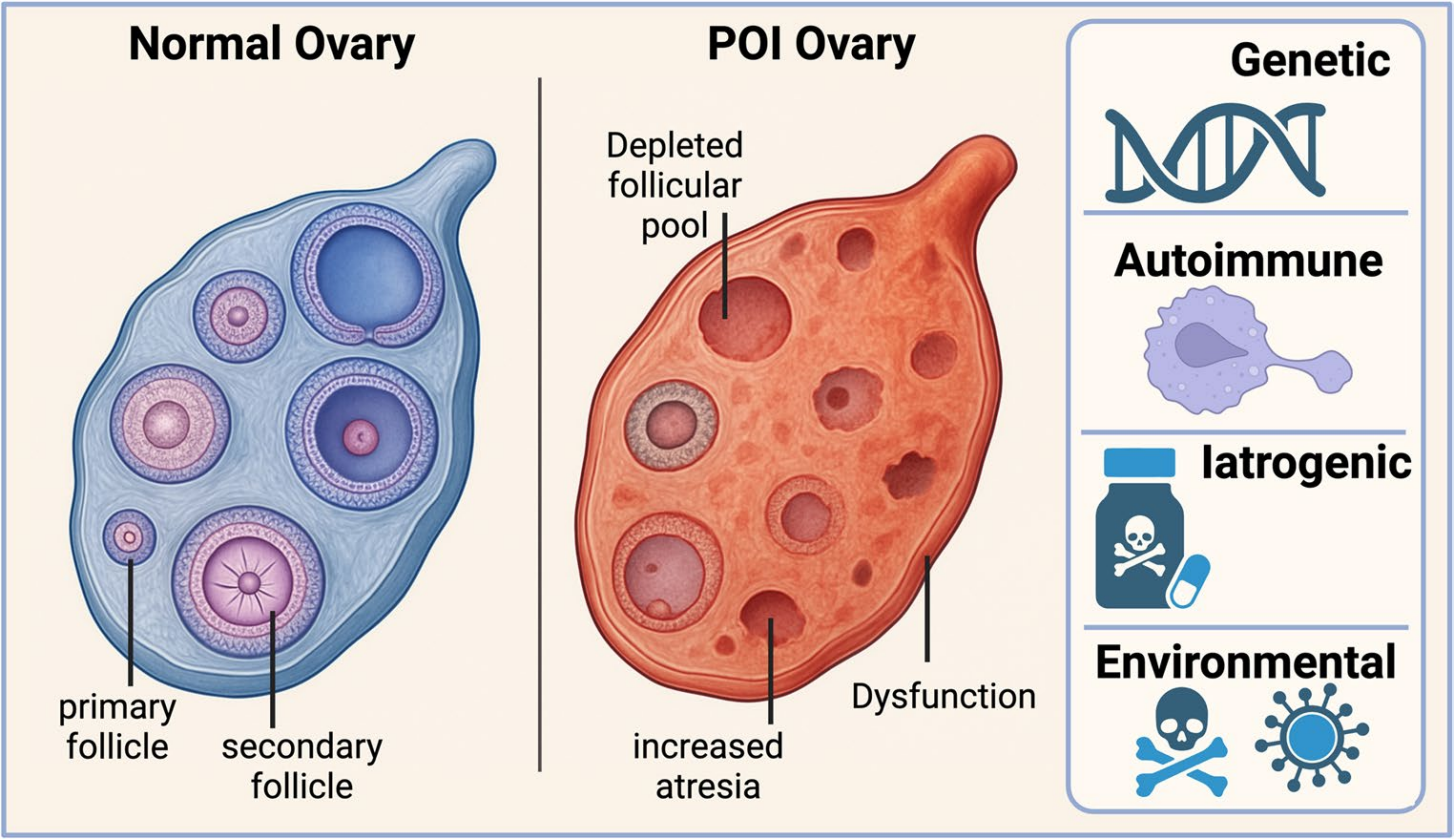
Pathophysiology of Premature ovarian insufficiency (POI). The figure shows the difference between a normal ovary and POI. The normal ovary has the potential to form active primordial follicles while in POI the ovary loses all primordial follicles (early eggs). Adopted from [30] (Created in https://BioRender.com)

Premature ovarian insufficiency (POI) is a heterogeneous disorder characterized by the loss of ovarian activity before the age of 40 (Fig. 1) [19]. While the clinical presentation often involves amenorrhea or oligomenorrhea, hypoestrogenism, and elevated gonadotropin levels (FSH > 25–40 IU/L on two occasions at least 4 weeks apart), the underlying pathophysiology is complex and involves a premature depletion of the ovarian follicular pool or dysfunction of existing follicles (Fig. 1) [20, 21].

Normally, a woman is born with a finite number of primordial follicles, which gradually decline throughout her reproductive life until menopause [22, 23]. In POI, this process is drastically accelerated. The mechanisms leading to POI can be broadly categorized as [24, 25] (1) Accelerated follicular atresia, which is the most common underlying mechanism, where the rate of follicular death (atresia) is significantly increased, leading to a rapid exhaustion of the ovarian reserve. Factors contributing to accelerated atresia include genetic defects, autoimmune processes, and exposure to gonadotoxic agents [26, 27]. (2) Follicular Dysfunction: In some cases, follicles may be present in the ovaries but fail to respond appropriately to gonadotropin stimulation or to mature properly. This can be due to defects in gonadotropin receptors, signaling pathways, or oocyte-granulosa cell communication [28, 29].

#### Etiological Factors

The etiology of POI is diverse and often multifactorial. In a large percentage of cases (up to 75–90% in some series), the cause remains idiopathic (unknown) despite extensive investigation [27]. However, several known causes have been identified as shown in Table 1.

**Table 1:** Etiological factors of premature ovarian insufficiency

Genetic Factors: These account for a significant proportion of known causes [24].	
Chromosomal Abnormalities	Turner syndrome (45, X and its variants) is a classic example. X chromosome deletions, translocations, or mosaicism can also lead to POI [31].
Single Gene Mutations	Mutations in numerous genes involved in ovarian development, follicle maturation, meiosis, DNA repair, and hormone synthesis/action have been implicated. Examples include mutations in FMR1 (Fragile X premutation), BMP15, GDF9, NOBOX, FIGLA, FSHR, LHCGR, NR5A1 (SF1), and genes involved in DNA repair pathways (e.g., BRCA1, BRCA2, MCM8, MCM9) [32, 33].
Autoimmune Disorders: Autoimmune oophoritis, where the immune system mistakenly attacks ovarian tissue (targeting oocytes, granulosa cells, or theca cells), can lead to POI. This can occur as an isolated condition or in association with other autoimmune diseases, such as autoimmune thyroiditis, Addison’s disease, type 1 diabetes mellitus, or systemic lupus erythematosus. The presence of anti-ovarian antibodies or lymphocytic infiltration in ovarian biopsies supports this diagnosis, though antibody testing has limitations in sensitivity and specificity [34, 35].	
Iatrogenic Causes: Medical treatments can inadvertently damage the ovaries [36–38].	
Chemotherapy	Alkylating agents are particularly gonadotoxic, but other chemotherapeutic drugs can also impair ovarian function.
Radiotherapy	Pelvic irradiation can destroy ovarian follicles, with the extent of damage depending on the radiation dose and the patient’s age.
Ovarian Surgery	Procedures such as bilateral oophorectomy, or even surgeries for benign conditions like endometriomas, can reduce ovarian reserve or compromise blood supply, potentially leading to POI.
Environmental Factors and Infections [39, 40]:	
Toxins	Exposure to certain environmental toxins, such as cigarette smoke, pesticides, and industrial chemicals, has been linked to ovarian damage and earlier menopause, potentially contributing to POI in susceptible individuals.
Infections	Viral infections like mumps oophoritis (though rare with vaccination) have been implicated. The role of other infections is less clear but remains an area of research.
Metabolic Disorders: Galactosemia, a rare metabolic disorder, can lead to POI if not treated early with a galactose-restricted diet [41].	

However, the mechanisms by which MSCs exert their therapeutic effects in POI are multifaceted and extend beyond this single pathway [42, 43] (Table 2).

**Table 2:** Therapeutic mechanisms of mesenchymal stem cells in premature ovarian insufficiency

Paracrine Signalling	MSCs secrete a wide array of growth factors (e.g., VEGF, HGF, IGF-1, FGF2), cytokines (e.g., IL-6, IL-10), and chemokines that can promote cell survival, proliferation, angiogenesis, and modulate immune responses within the ovarian microenvironment [44].
Immunomodulation	MSCs can suppress pro-inflammatory responses and promote an anti-inflammatory milieu, which is beneficial in cases of autoimmune oophoritis or chemotherapy-induced ovarian inflammation [42].
Anti-apoptotic Effects	MSCs can protect ovarian cells, particularly granulosa cells, from apoptosis induced by various stressors [45, 46].
Anti-fibrotic Effects	MSCs may reduce ovarian fibrosis, which can impair follicular development and ovarian function [47].
Mitochondrial Transfer	There is emerging evidence that MSCs can transfer healthy mitochondria to damaged cells, potentially restoring cellular function [48, 49].
Extracellular Vesicle (EV) Secretion	MSC-derived EVs, especially exosomes, carry proteins, lipids, mRNAs, and microRNAs that can mediate many of the paracrine effects of MSCs, offering a potential cell-free therapeutic approach [50–52].

Understanding these diverse mechanisms is critical for optimizing MSC-based therapies for POI and will be explored in more detail in subsequent sections.

### Very Small Embryonic-Like Stem Cells (VSELs) in Ovaries

Recent research has identified the presence of very small embryonic-like stem cells (VSELs) in adult mammalian ovaries, which may have significant implications for understanding and treating POI [53, 54]. VSELs are small (3–6 μm) pluripotent stem cells that express markers of pluripotency such as OCT-4 A, SSEA-1, and NANOG.

Unlike the traditional understanding that female mammals are born with a fixed number of oocytes that cannot be replenished, evidence suggests that VSELs may represent a population of stem cells in the ovarian surface epithelium that can generate new oocytes throughout reproductive life [55, 56]. These cells remain relatively quiescent under normal conditions but can be activated in response to injury or disease.

Studies have demonstrated that VSELs survive chemotherapy in mouse ovaries while more mature follicular cells are destroyed [57]. This observation has important implications for fertility preservation and POI treatment, as it suggests that the ovary retains regenerative potential even after chemotherapy-induced damage. Stimulating the surviving VSELs could potentially restore ovarian function and fertility [58, 59].

Furthermore, research indicates that VSELs may interact with ovarian somatic cells (particularly the ovarian surface epithelium) to initiate follicular development [37, 60]. This interaction appears to be regulated by various growth factors and cytokines, some of which are also secreted by MSCs. This suggests a potential synergistic relationship between endogenous VSELs and transplanted MSCs in ovarian regeneration [15, 43, 61].

The discovery of VSELs challenges the central dogma of fixed ovarian reserve and opens new avenues for POI treatment. Future research focusing on methods to activate endogenous VSELs, possibly in combination with MSC therapy, may lead to more effective regenerative approaches for women with POI [62, 63].

### Mesenchymal Stem Cell-Based Fertility Restoration in POI: Mechanisms and Evidence

**Fig. 2:**
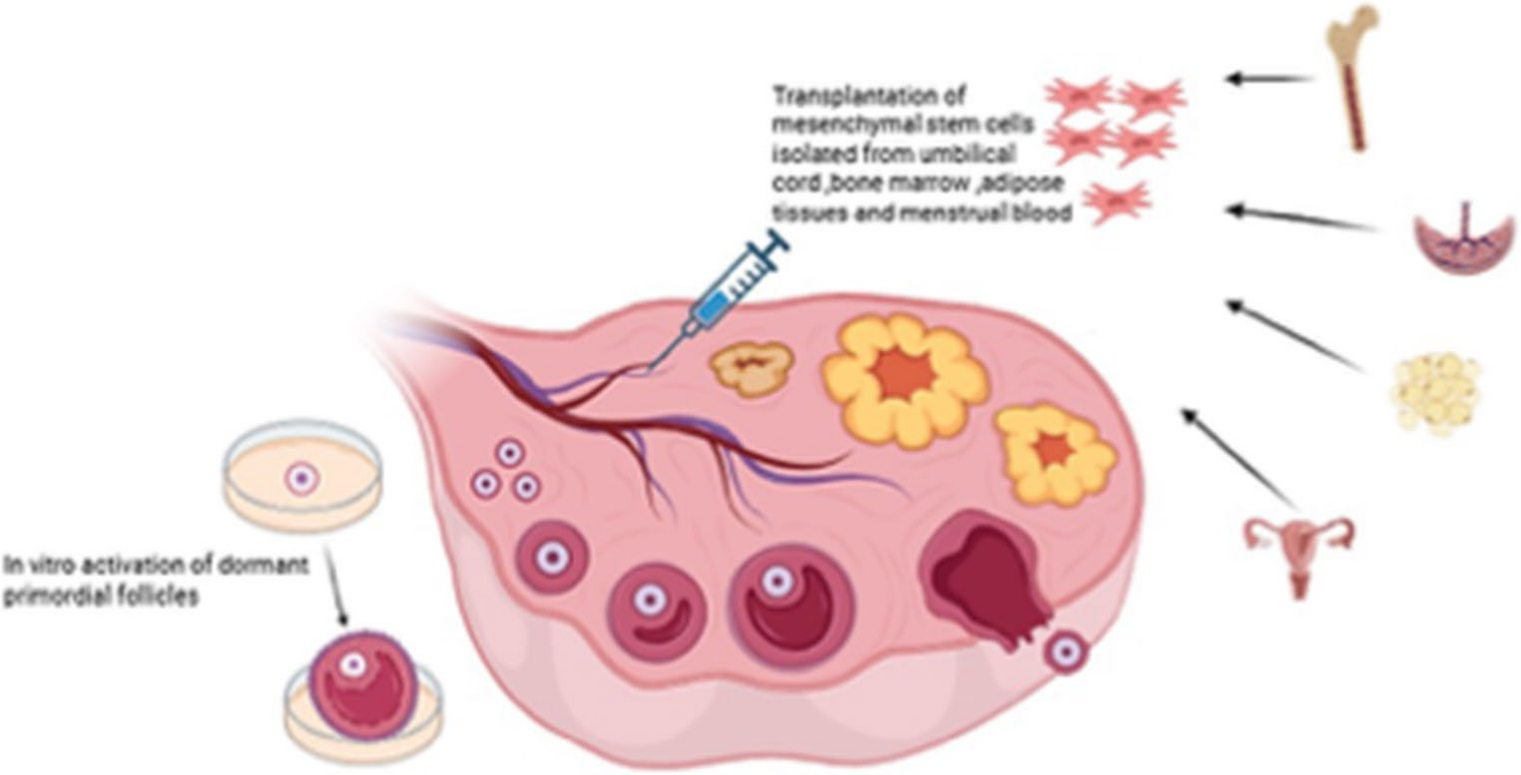
The therapeutic potential of mesenchymal stem cells isolated from different sources through their transplantation in dysfunctional ovaries due to POI in vivo and through activation of dormant primordial follicles in vitro in POI treatment. Adopted from [66]. Created in https://BioRender.com

The therapeutic potential of mesenchymal stem cells (MSCs) in restoring fertility for individuals with premature ovarian insufficiency (POI) represents one of the most dynamic and promising frontiers in regenerative medicine (Fig. 2) [13]. Conventional treatments for POI-related infertility, such as hormone replacement therapy (HRT) and assisted reproductive technologies (ART) with oocyte donation, address symptoms or bypass ovarian dysfunction but do not restore endogenous ovarian function [13, 64]. MSC-based therapies, in contrast, aim to rejuvenate the ovarian microenvironment, protect existing follicles, and potentially stimulate the activation of dormant primordial follicles, thereby offering a chance for natural conception or improved response to ART using autologous oocytes [13, 65].

### Sources of Mesenchymal Stem Cells for POI Therapy

**Fig. 3:**
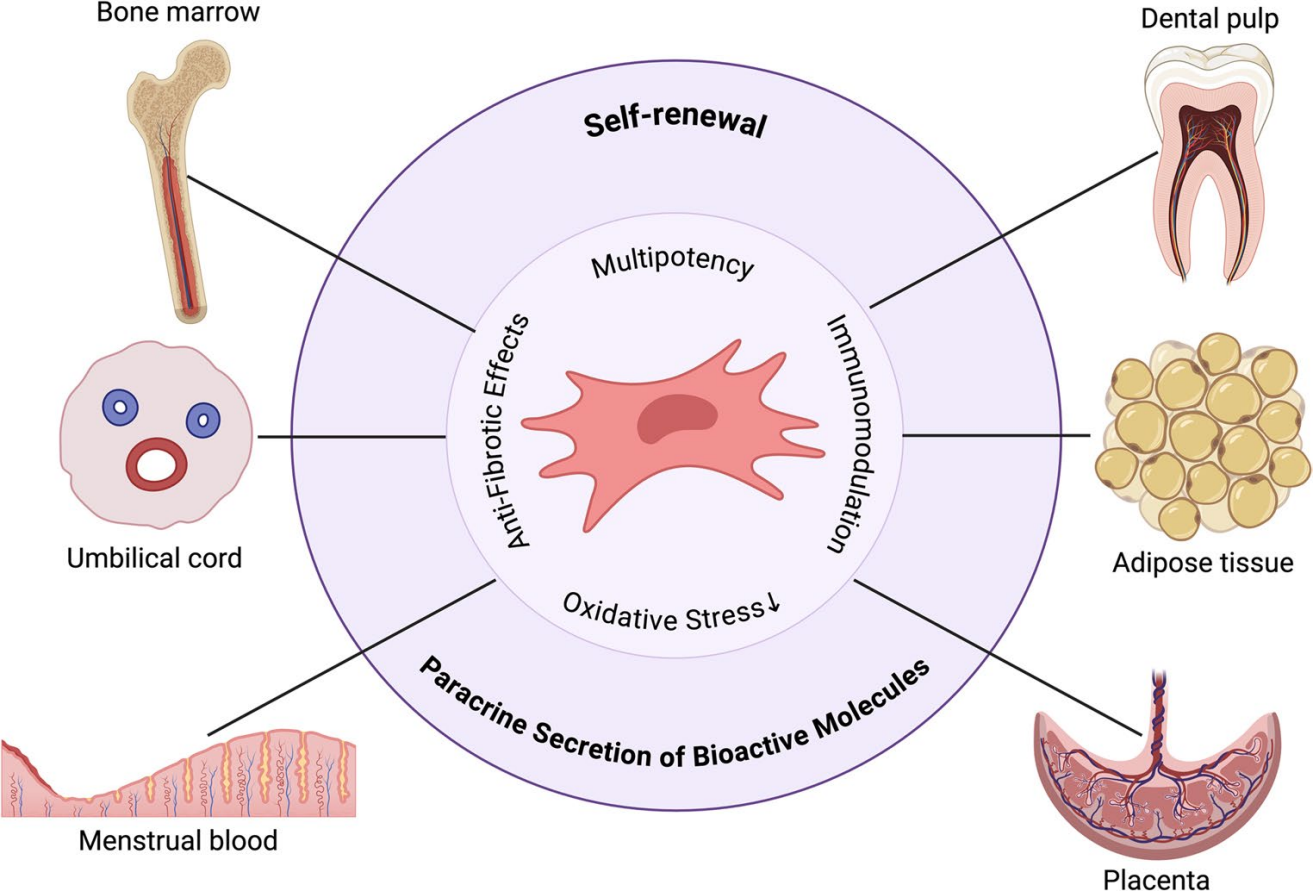
Sources of Mesenchymal Stem Cells for POI Treatment. Adopted from [76]. Created in https://BioRender.com

MSCs can be isolated from various adult and perinatal tissues, each with its own set of advantages and disadvantages for therapeutic application in POI (Fig. 3) [67]:

- ***Bone Marrow-Derived MSCs (BM-MSCs)***: Historically the most studied source, BM-MSCs have well-characterized regenerative properties. However, their isolation requires an invasive procedure, and their proliferation capacity and differentiation potential may decline with donor age [68].
- ***Adipose-Derived MSCs (AD-MSCs)***: AD-MSCs are abundant and can be obtained through a less invasive liposuction procedure. They exhibit robust proliferative capacity and potent immunomodulatory and angiogenic effects, making them an attractive option for POI [69].
- ***Umbilical Cord-Derived MSCs (UC-MSCs)***: These include MSCs from Wharton’s jelly (WJ-MSCs) and umbilical cord blood. UC-MSCs are considered more primitive, possess higher proliferation rates, lower immunogenicity, and potent paracrine activity compared to adult MSCs. Their collection is non-invasive and ethically straightforward [70]. Several studies have highlighted the particular efficacy of UC-MSCs in POI models [71].
- ***Placenta-Derived MSCs (P-MSCs)***: The placenta is another rich source of young, highly proliferative MSCs with strong immunomodulatory properties. Similar to UC-MSCs, their collection is non-invasive [72].
- ***Menstrual Blood-Derived MSCs (MenSCs)***: MenSCs can be easily and repeatedly collected non-invasively. They have shown promise in various regenerative applications, including endometrial regeneration and potentially ovarian rejuvenation, though research in POI is still emerging [73].
- ***Other Sources***: MSCs have also been isolated from amniotic fluid, dental pulp, and induced pluripotent stem cells (iPSC-MSCs). iPSC-MSCs offer the potential for autologous therapy without invasive harvesting from adult tissues, but their generation and clinical translation face challenges related to safety and standardization [74, 75].

The choice of MSC source may influence therapeutic outcomes, and further research is needed to determine the optimal source for POI treatment.

### Mechanisms of MSC-Mediated Ovarian Restoration

MSCs exert their therapeutic effects in POI through a complex interplay of mechanisms, primarily driven by their paracrine activity rather than direct differentiation into ovarian cell types, which remains controversial and unlikely to be a major contributor to functional recovery [60, 77].

- ***Paracrine Secretion of Bioactive Molecules***: MSCs release a plethora of growth factors (e.g., vascular endothelial growth factor (VEGF), hepatocyte growth factor (HGF), insulin-like growth factor 1 (IGF-1), basic fibroblast growth factor (bFGF)), cytokines (e.g., interleukin-10 (IL-10) and transforming growth factor-beta (TGF-β)), and chemokines [43, 44, 78, 79]. These factors collectively promote angiogenesis, where VEGF and bFGF stimulate the formation of new blood vessels, improving ovarian perfusion and nutrient supply, which is crucial for follicular survival and development [80]. They also inhibit apoptosis, where MSC-secreted factors can protect granulosa cells and oocytes from programmed cell death, a key feature of follicular atresia in POI [81], and stimulate cell proliferation and survival via growth factors like HGF and IGF-1, which can support the proliferation and survival of ovarian stromal and follicular cells [82, 83].
- ***Immunomodulation***: MSCs possess potent immunomodulatory capabilities. They can suppress the activity of pro-inflammatory immune cells (e.g., Th1, Th17 lymphocytes, M1 macrophages) and promote an anti-inflammatory environment by inducing regulatory T cells (Tregs) and M2 macrophages. This is particularly relevant for autoimmune POI and for mitigating inflammation associated with chemotherapy-induced ovarian damage [42, 51, 72, 84].
- ***Anti-Fibrotic Effects***: Chronic inflammation and tissue damage in POI can lead to ovarian fibrosis, impairing follicular development. MSCs can secrete anti-fibrotic factors and enzymes that degrade excess extracellular matrix, potentially reversing or limiting ovarian fibrosis [47, 85].
- ***Reduction of Oxidative Stress***: Oxidative stress is a significant contributor to oocyte aging and follicular damage in POI. MSCs can enhance the antioxidant capacity of ovarian tissue by secreting antioxidant enzymes or by upregulating endogenous antioxidant pathways in ovarian cells [86, 87].
- ***Mitochondrial Transfer***: Emerging evidence suggests that MSCs can transfer healthy mitochondria to damaged ovarian cells via tunneling nanotubes or extracellular vesicles. This mitochondrial donation can rescue cells with mitochondrial dysfunction, improve cellular energy metabolism, and reduce apoptosis [48, 49].
- ***Activation of Dormant Primordial Follicles***: While the exact mechanisms are still being elucidated, MSCs may promote the activation of the remaining pool of dormant primordial follicles, potentially through the secretion of factors that influence the PI3K/AKT/mTOR pathway or other signaling cascades involved in follicle awakening [81, 88, 89].

### Preclinical and Clinical Evidence

Numerous preclinical studies using various animal models of POI (induced by chemotherapy, autoimmune mechanisms, or genetic factors) have demonstrated the efficacy of MSC transplantation from different sources [60, 81]. These studies consistently show improvements in ovarian morphology, increased numbers of healthy follicles at different developmental stages, restoration of hormone levels (e.g., increased estrogen and anti-Müllerian hormone (AMH), decreased FSH), reduced granulosa cell apoptosis, enhanced angiogenesis, and, in many cases, restoration of fertility with successful pregnancies [13, 80]. For instance, a recent meta-analysis of preclinical studies confirmed significant improvements in ovarian function markers following MSC therapy [15, 90].

Clinical translation of MSC therapy for POI is still in its early stages, but initial results from pilot studies and small clinical trials are encouraging. These studies have generally reported good safety and tolerability of MSC administration (often via intraovarian injection or systemic infusion) [66, 91, 92]. Reported outcomes in some patients include improvements in hormonal profiles, increased antral follicle counts, resumption of menstruation, and even spontaneous pregnancies [93, 94]. However, these trials are often limited by small sample sizes, lack of control groups, variability in MSC sources, dosage, and delivery methods, and short follow-up periods. Larger, well-controlled randomized clinical trials are crucial to definitively establish the efficacy and safety of MSC therapy for POI in humans [95, 96].

### The Emerging Role of MSC-Derived Extracellular Vesicles (Exosomes) in POI Therapy

**Fig. 4:**
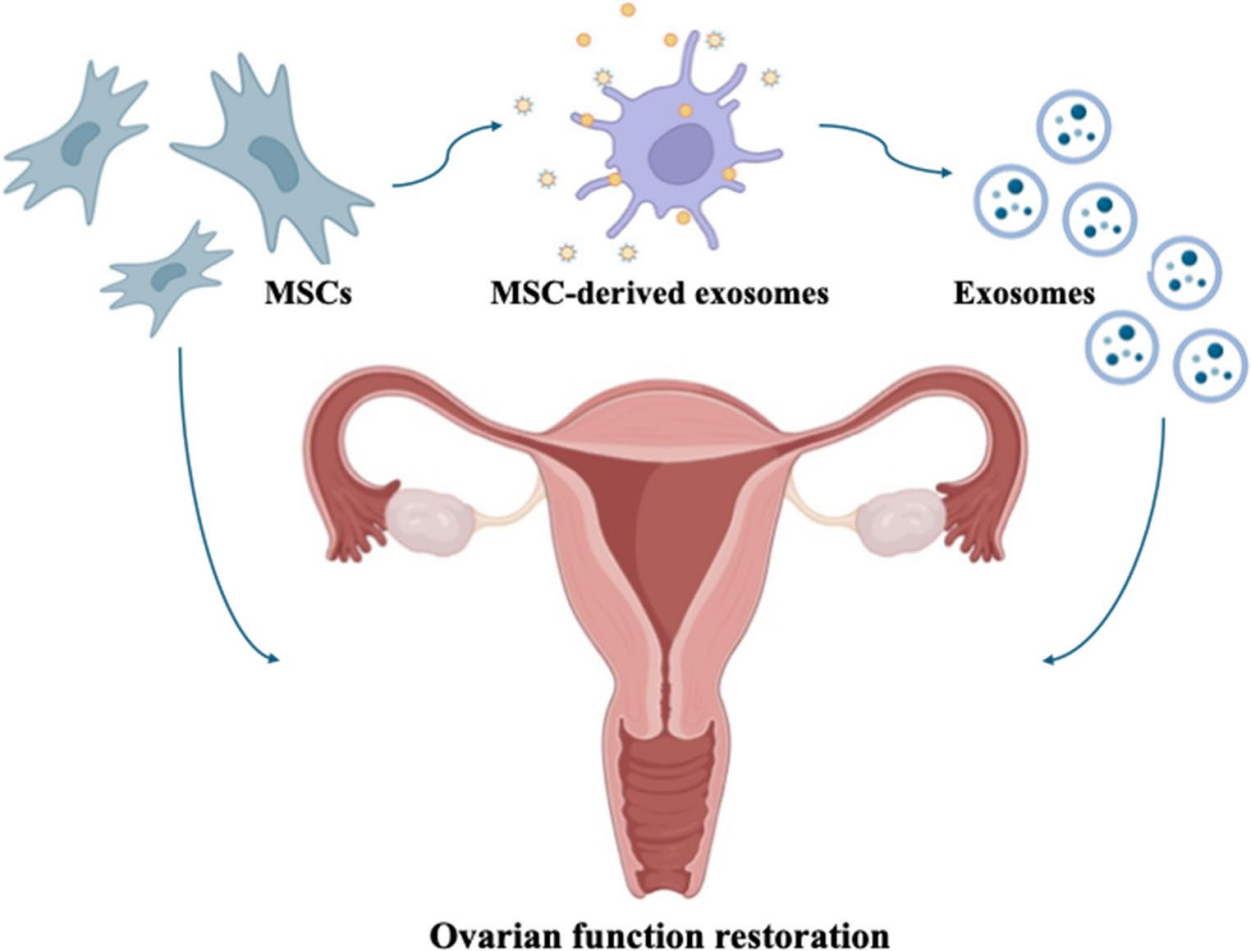
MSCs stem cell-derived exosomes in POI treatment. Exosomes have the potential to markedly increase ovarian function and reproductive capacity in POI through stimulation of granulosa cells (GCs) inside the ovary. Adopted from [13] (created in https://BioRender.com)

In recent years, the therapeutic potential of mesenchymal stem cell (MSC)-derived extracellular vesicles (EVs), particularly exosomes, has garnered significant attention as a novel cell-free approach for treating various diseases, including premature ovarian insufficiency (POI) (Fig. 4) [97–99]. Exosomes are nano-sized (typically 30–150 nm) membrane-bound vesicles secreted by most cell types, including MSCs [98, 100]. They act as intercellular messengers, transferring a diverse cargo of bioactive molecules—such as proteins, lipids, mRNAs, and microRNAs (miRNAs)—from their parent cells to recipient cells, thereby modulating the function of the recipient cells [101, 102].

### Exosome-Based Therapy Over Whole-Cell MSC Therapy

Using MSC-derived exosomes instead of whole MSCs offers several potential advantages, including an improved safety profile, knowing that exosomes are non-living and cannot replicate, eliminating risks associated with whole-cell therapy, such as uncontrolled proliferation, differentiation into undesirable cell types, or tumorigenicity (though the risk with MSCs themselves is very low) [103, 104]. Exosomes generally exhibit lower immunogenicity compared to their parent cells, potentially allowing for repeated administrations without eliciting strong immune responses [105, 106]. Exosomes can also be more easily stored and transported than live cells, facilitating off-the-shelf therapeutic products [107, 108]. Furthermore, characterizing and standardizing exosome preparations may be more straightforward than for complex cellular products [109]. Due to their small size, exosomes may be better able to penetrate tissues and cross biological barriers [110].

#### Mechanisms of Action of MSC-Exosomes in POI

MSC-derived exosomes are believed to mediate many of the regenerative effects previously attributed to MSCs themselves [98]. In the context of POI, MSC-exosomes have been shown in preclinical studies to promote granulosa cell proliferation and inhibit apoptosis, where exosomal cargo, particularly specific miRNAs and growth factors, can protect granulosa cells from damage and support their function [111]. Exosomes can also deliver pro-angiogenic factors (e.g., VEGF) or miRNAs that promote neovascularization in the ovary, improving blood supply and follicular health [112]. MSC-exosomes can carry immunomodulatory molecules that suppress inflammation and promote a tolerogenic microenvironment in the ovary [113, 114]. Exosomes may transfer antioxidant enzymes or molecules that enhance the antioxidant capacity of ovarian cells [115]. On the other hand, specific miRNAs within exosomes (e.g., miR-17-5p, miR-146a, miR-21) have been implicated in regulating follicular growth, atresia, and steroidogenesis. For example, studies have shown that MSC-exosomes can upregulate AMH expression and promote the transition from primordial to primary follicles [116–118]. By delivering a cocktail of beneficial molecules, exosomes can help restore homeostasis to the damaged ovarian microenvironment [119, 120].

#### Preclinical Evidence for MSC-Exosomes in POI

A growing body of preclinical research supports the therapeutic potential of MSC-exosomes in POI [121, 122]. Studies using animal models of chemotherapy-induced or age-related ovarian dysfunction have demonstrated that administration of MSC-exosomes can lead to **(1)** restoration of ovarian function, including regular estrous cycles and improved hormone levels (e.g., increased estrogen and AMH, decreased FSH) [123] **(2)** increased number of healthy follicles and reduced follicular atresia [124]; **(3)** enhanced ovarian angiogenesis and reduced ovarian fibrosis [141]; and **(4)** improved oocyte quality and even successful pregnancies after exosome treatment in some models [124].

#### Challenges and Future Directions for Exosome Therapy in POI

Despite the promising preclinical data, several challenges need to be addressed before MSC-exosome therapy can become a clinical reality for POI, including **(1)** developing standardized and scalable methods for isolating and characterizing exosomes is crucial for ensuring product consistency and quality [125], **(2)** identifying the specific therapeutic components within the exosomal cargo and developing assays to measure their potency are ongoing research areas [126], **(3)** determining the optimal dose, delivery route (e.g., systemic vs. intraovarian injection), and timing of exosome administration requires further investigation [127], and establishing cost-effective methods for large-scale production of clinical-grade exosomes is necessary for widespread application [128]. Moreover, rigorous, well-designed clinical trials are needed to evaluate the safety and efficacy of MSC-exosome therapy in women with POI [71]. Nevertheless, MSC-derived exosomes represent a highly promising next-generation, cell-free therapeutic strategy for POI, potentially offering a safer and more practical alternative to whole-cell therapies.

## Challenges, Future Directions, and Conclusion

### Challenges in Translating MSC-Based Therapies for POI To the Clinic

Despite the promising preclinical data and early clinical observations, several challenges must be overcome to successfully translate MSC-based therapies (including cell-based and cell-free approaches like exosomes) into routine clinical practice for POI [129]. Standardization of MSC manufacturing remains a significant hurdle, with considerable variability in isolation protocols, culture conditions (e.g., use of fetal bovine serum vs. serum-free media, 2D vs. 3D culture), characterization methods, and expansion procedures, necessitating the establishment of standardized, Good Manufacturing Practice (GMP)-compliant protocols to ensure product consistency, safety, and efficacy [130–133]. The optimal cell source (bone marrow, adipose, umbilical cord, etc.), dosage, delivery route (e.g., systemic intravenous infusion, direct intraovarian injection via laparoscopy or ultrasound guidance), and timing and frequency of treatment are yet to be definitively established, with intraovarian injection offering targeted delivery but being more invasive than systemic routes, highlighting the need for comparative studies to address these critical parameters [134–136]. Long-term data on the durability of therapeutic effects and potential late adverse events (e.g., immunogenicity with repeated doses, ectopic tissue formation, though rare for MSCs) are crucial, as most clinical studies to date have relatively short follow-up periods [125, 129]. Identifying the subset of POI patients most likely to benefit from MSC therapy is important [137], with early-onset POI (before age 40) and POI with preserved ovarian tissue potentially more responsive to treatment; factors such as age, etiology of POI, and remaining ovarian reserve may influence outcomes, while standardized, clinically meaningful efficacy measures (e.g., sustained restoration of menses, hormonal balance, antral follicle count, oocyte quality, live birth rates) need to be consistently applied across trials [138–140]. A more precise understanding of the specific molecular mediators (e.g., key growth factors, cytokines, exosomal miRNAs) and signaling pathways involved in MSC-mediated ovarian repair is needed to optimize therapies and develop targeted interventions, despite the wide acceptance of paracrine effects [43, 141]. Finally, evaluating the cost-effectiveness of these potentially expensive treatments compared to existing options will be important for their broader adoption [142, 143].

### Future Directions in MSC Research for POI

The field of MSC research for POI is rapidly evolving, with several exciting future directions. Advanced MSC engineering, including genetic modification to overexpress specific therapeutic factors (e.g., anti-inflammatory cytokines, pro-angiogenic factors) or to enhance ovarian homing, could improve efficacy, while preconditioning MSCs under hypoxic conditions may boost their paracrine activity [144, 145]. Cell-free therapies using MSC-derived exosomes or other EVs hold immense promise due to their potential safety, stability, and manufacturing advantages, with research focusing on optimizing exosome production, characterizing therapeutic cargo, and conducting clinical trials [145]. Combination therapies that merge MSC treatment with antioxidants, growth factors, or existing ovarian stimulation protocols may yield synergistic effects [146]. The use of biocompatible scaffolds to deliver MSCs or their products directly to the ovary could enhance retention, survival, and local therapeutic effects, potentially creating a regenerative ovarian niche [147–149]. Personalized medicine approaches that tailor MSC therapies based on the specific etiology of POI in individual patients or their genetic background may improve outcomes [150]. While not a direct MSC therapy, advances in creating ovarian organoids from iPSCs or other cell sources, potentially supported by MSCs or their secretome, could offer future avenues for understanding ovarian biology and developing novel fertility restoration techniques [151, 152]. Finally, developing non-invasive methods to track MSCs post-transplantation and to monitor ovarian responses to therapy will be valuable for optimizing treatment protocols [153].

## Conclusion

Premature ovarian insufficiency is a challenging condition with profound implications for female reproductive and overall health [3, 20]. Current management strategies are largely supportive, failing to address the underlying loss of ovarian function [20]. Mesenchymal stem cell therapy, leveraging the unique regenerative and immunomodulatory properties of MSCs, has emerged as a highly promising therapeutic avenue [15, 42, 84]. A wealth of preclinical evidence demonstrates the ability of MSCs, and increasingly their derived exosomes, to improve ovarian function, enhance folliculogenesis, and restore fertility in various POI models [109, 145]. These effects are mediated through complex paracrine mechanisms, including the secretion of growth factors, cytokines, and miRNAs that collectively reduce inflammation and apoptosis, promote angiogenesis, modulate the immune system, and improve the ovarian microenvironment [43, 78, 122].

Early clinical trials have provided encouraging, albeit preliminary, evidence of the safety and potential efficacy of MSC therapy in women with POI [27, 121]. However, the field is still in its nascent stages, and significant challenges related to standardization, optimal treatment protocols, long-term outcomes, and regulatory approval must be addressed.

Future research focused on elucidating the precise mechanisms of action, optimizing MSC sources and delivery methods, exploring the potential of engineered MSCs and cell-free exosome-based therapies, and conducting rigorous, large-scale clinical trials will be critical for translating this promising therapeutic modality into a widely available and effective treatment for POI. The continued exploration of MSCs and their derivatives offers tangible hope for restoring ovarian function and improving the quality of life for the many women affected by this debilitating condition, moving beyond symptomatic relief towards true ovarian rejuvenation and fertility restoration.

## Data Availability

No datasets were generated or analysed during the current study.
